# Serum metabolomic profiling reveals an increase in homocitrulline in Chinese patients with nonalcoholic fatty liver disease: a retrospective study

**DOI:** 10.7717/peerj.11346

**Published:** 2021-05-03

**Authors:** Yarong Yang, Zexin Huang, Zhao Yang, Ying Qi, Hui Shi, Yifei Zhou, Fangyu Wang, Miaofang Yang

**Affiliations:** 1 Department of Gastroenterology and Hepatology, Jinling Hospital, the First School of Clinical Medicine, Southern Medical University, Nanjing, Jiangsu, China; 2 Department of Gastroenterology and Hepatology, Jinling Hospital, Medical School of Nanjing University, Nanjing, Jiangsu, China

**Keywords:** Glycerophospholipids, Homocitrulline, Non-alcoholic fatty liver disease (NAFLD), Metabolomics, Metabolic pathways

## Abstract

**Backgrounds:**

Nonalcoholic fatty liver disease (NAFLD) has multiple causes, is triggered by individual genetic susceptibility, environmental factors, and metabolic disturbances, and may be triggered by acquired metabolic stress. The metabolic profiles of NAFLD show significant ethnic differences, and the metabolic characteristics of NAFLD in Chinese individuals are unclear. Our study aimed to identify the metabolites and pathways associated with NAFLD in a Chinese cohort.

**Methods:**

One hundred participants, including 50 NAFLD patients and 50 healthy controls, were enrolled in this retrospective observational study at Jinling Hospital in Nanjing; serum samples were collected from the patients and healthy subjects. The metabolome was determined in all samples by liquid chromatography-hybrid quadrupole time-of-flight mass spectrometry (LC-Q/TOF-MS). Univariate and multivariate statistical analyses were used to compare the metabolic profiles between the two groups.

**Results:**

The comparison indicated that the levels of 89 metabolites were different between the two groups. The glycerophospholipid family of metabolites was the most abundant family of metabolites that demonstrated significant differences. L-acetylcarnitine, L-homocitrulline, and glutamic acid were the top three metabolites ranked by VIP score and had favorable effective functions for diagnosis. Moreover, pathway enrichment analysis suggested 14 potentially different metabolic pathways between NAFLD patients and healthy controls based on their impact value. Biological modules involved in the lipid and carbohydrate metabolism had the highest relevance to the conditions of NAFLD. Glycerophospholipid metabolism had the strongest associations with the conditions of NAFLD.

**Conclusions:**

Our data suggest that the serum metabolic profiles of NAFLD patients and healthy controls are different. L-Homocitrulline was remarkably increased in NAFLD patients.

## Introduction

NAFLD is a clinicopathological condition defining a spectrum of liver diseases, including nonalcoholic fatty liver (NAFL), nonalcoholic steatohepatitis (NASH), cirrhosis, and hepatocellular carcinoma (Brunt, 2010; Brunt et al., 2015; Fan et al., 2019). NAFLD is associated with inefficient nutrient metabolism and usually develops in the context of metabolic syndrome (MetS) (Friedman et al., 2018; Yki-Järvinen, 2014). Currently, the prevalence of NAFLD in most Asian countries remains over 25%, making it the most common chronic liver disease (Younossi et al., 2016).

Metabolomics is used to analyze the profiles of small molecule metabolites of cellular processes (Nicholson et al., 2002). Currently, metabolomics is used for disease prediction, differential diagnosis, drug response assessment, and hypothesis generation (Di Dalmazi et al., 2017; Soga et al., 2011; Tzoulaki et al., 2014; Yin & Xu, 2014). A number of studies have demonstrated differences in the metabolomic profiles and some crucial metabolic pathways between NAFLD patients and healthy controls in other countries (Fellinger et al., 2020; Gaggini et al., 2018; Gitto et al., 2018; Gorden et al., 2015; Takahashi et al., 2020; Tang et al., 2019). In China, a variety of studies have concentrated on the effects and mechanisms of action of medicines used to alleviate NAFLD (Deng et al., 2019; Tanaka et al., 2017; Wang et al., 2016) However, only a few published studies have focused on the metabolic profiles of NAFLD patients in China, and the results of metabolomic studies in NAFLD are inconsistent. We aimed to analyze the metabolomic profiles of Chinese NAFLD patients.

Nontargeted liquid chromatography-mass spectrometry with quadrupole time-of-flight mass spectrometry (LC-Q-TOF/MS) was used for the analysis of the serum to provide the data to identify altered endogenous metabolites and pathways associated with NAFLD in a Chinese cohort enrolled in this study. We sought to evaluate whether LC-MS analysis can distinguish NAFLD patients from healthy controls based on differential metabolic profiles. Then, the alterations in the metabolites and related pathways were defined to explain the mechanism of NAFLD.

## Materials & Methods

### Study population and sample Collection

One hundred subjects, including 50 healthy controls and 50 NAFLD patients admitted to the outpatient clinic at Jinling Hospital in Nanjing, China, were enrolled in this study from January 2015 to December 2018. The inclusion criteria for the NAFLD group were as follows: (1) hepatic steatosis diagnosed by imaging; (2) age between 14 and 75 years; and (3) history of alcohol consumption of <210 g/week in men and <140 g/week in women over 2 years prior to the diagnosis of hepatic steatosis. The exclusion criteria for the NAFLD group were as follows: (1) positive results of a serum test for hepatitis B virus surface antigen and hepatitis C virus antibodies; (2) patients with alcoholic liver disease, drug-induced liver injury, total parenteral nutrition, hepatolenticular degeneration, autoimmune liver disease, and other specific diseases that can cause fatty liver (Chalasani et al., 2012); (3) patients who have taken nonsteroidal anti-inflammatory drugs, anticoagulants, antibiotics, and proton pump inhibitors in the past month; (4) patients who have lost weight through diet or vigorous exercise within the past month; (5) patients with serious diseases, such as heart, lung, brain, or kidney diseases; and (6) patients with malignant tumors or autoimmune diseases.

Blood samples were collected from the enrolled subjects after at least 12 h of fasting. Serum samples were obtained by centrifugation (3,500 rpm, 6 min) and divided into two parts; one part was stored at −80 °C until analysis, and another part was used for the detection of albumin, aspartate aminotransferase (AST), alanine aminotransferase (ALT), uric acid, triglycerides, fasting blood glucose, and total cholesterol at Nanjing Jinling Hospital Laboratory by a Hitachi 7600-110 automatic biochemical analyzer (Hitachi, Tokyo, Japan).

### Serum sample pretreatment for LC-MS analysis

Before LC/MS analysis, 200 µL of serum samples, which were thawed at room temperature for 15 min, were mixed with 600 µL of methanol in the presence of 20 µg/mL DL-o-chlorophenyl alanine and vigorously vortexed for 30 s. The mixtures were centrifuged at 12,000 rpm for 15 min at 4 °C. A 200 µL aliquot of the supernatant was used for LC-MS analysis.

### LC-MS analysis

The samples were analyzed on an Agilent LC-Q/TOF-MS system (Agilent Technologies, Santa Clara, CA, USA), which consisted of an Agilent 1290 liquid chromatography system and an Agilent 6530 time-of-flight mass spectrometer. The samples were injected onto an Agilent C18 particle column (100 ×2.1 mm, 1.8 µm). The injected sample volume was 4 μL, and the flow rate was 0.35 mL/min. The column temperature was maintained at 45 °C. Solvent A consisted of 0.1% formic acid in water, and solvent B consisted of 0.1% formic acid in acetonitrile. The gradient of the mobile phase is shown in Table S1. An Agilent 6530 Accurate-Mass Q-TOF/MS (Agilent Technologies, CA, USA) equipped with an electrospray ionization (ESI) source in both negative mode and positive mode was used to perform the mass spectrometry assays. Nitrogen was used as a nebulizer gas. The measurement conditions were as follows: capillary voltage, −3.5 kV in ESI− and 4 kV in ESI+; sampling cone voltage, 50 kV in ESI− and 35 kV in ESI +; dissolving gas flow rate, 700 L/h in ESI− and 600 L/h in ESI+; source temperature, 100 °C in ESI− and ESI+; dissolving gas temperature, 350 °C in ESI− and ESI+; cone gas flow rate, 50 L/h in ESI− and ESI+; and extraction cone voltage, 4 kV in ESI− and ESI+. Centroid data were collected from 50 to 1,000 m/z, and the scan time was 0.03 s with an interscan delay of 0.02 s. The pooled quality control (QC) samples were used to ensure the stability and repeatability of the HPLC-Q-TOF system. QC was a mixture of 10 µl of each sample and was staggered after every ten samples; thus, the stability of the instrument could be investigated based on the overlap of QC chromatograms. The total ion current (TIC) chromatograms of the QC samples overlapped, as shown in Fig. S1.

### Statistical analysis

Two data sets from LC-QTOF/MS ESI+ and ESI− were used for peak selection, filtering, and filling by XCMS software. The differences in the metabolic features between the two groups were identified by the unsupervised method (principal component analysis, PCA) and supervised method (orthogonal partial least squares-discriminant analysis, OPLS-DA) using SIMCA-P software (Umetrics AB, Umea, Sweden). The Mann–Whitney U test was performed to identify differential metabolites between the two groups based on the false discovery rate (FDR). We matched the experimental tandem MS spectrum, retention time, and accurate mass of the metabolic features with spectral databases to identify the metabolites. Differential metabolites were characterized by search of an online database (HMDB) and comparison of mass spectra based on the mass-to-charge ratio or exact molecular mass. the SPSS software version 22.0 for Windows (SPSS, Inc., Chicago, IL, USA) was used to analyze the clinical data. Nonparametric data were analyzed using the Wilcoxon and Kruskal-Wallis tests and were expressed as the median with ranges, including ALT, AST, TG, TC, TBS and UA. Categorical data were analyzed using Fisher’s exact test, such as gender. Continuous variables, such as age, was analyzed using the two sample Student’s *t*-test and were expressed as mean ±standard deviation. MetaboAnalyst 4.0 (http://www.metaboanalyst.ca/) and Mbrole (http://csbg.cnb.csic.es/mbrole2/analysis.php) were used to perform metabolic pathway analysis. The significance level was set to a bilateral asymptotic *p*-value of <0.05.

### Ethics and consent

Informed consent was obtained from all individuals included in this study. Research involving human subjects was approved by the Institutional Review Board of Jinling Hospital (2014NZKY-007-01).

## Results

### Clinical characteristics of patients

**Figure 1:**
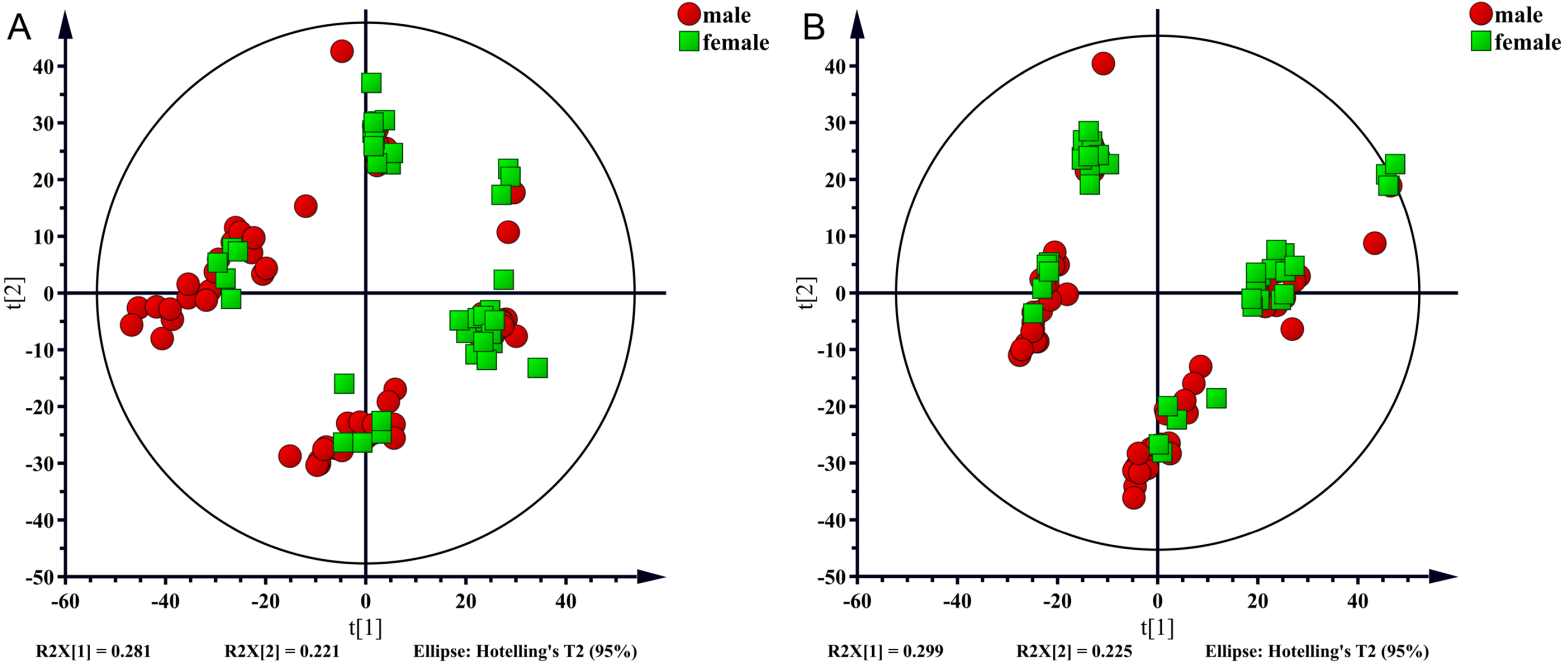
PCA score plot for males (red sopts) and females (green squares). (A) ESI+, (B) ESI−.

In the present study, 100 serum samples were analyzed using LC-MS to determine the metabolic profiles of 50 healthy controls and 50 NAFLD patients. The biochemical parameters are summarized in Table 1. There were no significant differences in age between patients and the control subjects (*p* = 0.304). The levels of serum triglycerides, total cholesterol, uric acid, AST, and ALT were significantly higher (*p* < 0.001) in the NAFLD group than those in the control group. The fasting blood glucose (FBG) level was higher in the NAFLD group (*p* < 0.001). The number of men was higher in the NAFLD group than in the control group (80% vs 42%, *p* < 0.001). We used unsupervised PCA to analyze associations of the serum metabolic profiles with sex. The results showed that metabolic profiles were similar in males and females which were shown in Fig. 1.

**Table 1:** Clinical characteristics of participants.

	NAFLD (*n* = 50)	Control (*n* = 50)	*P* value
Gender,M/F	40/10	21/29	**<0.001**^a^
AGE	39.54 ± 12.17	37.26 ± 9.75	0.304^b^
			*P*
ALT	44.5(23.75∼85.25)	15.7(12.88∼23.85)	**<0.001**^c^
AST	26(18.00∼38.75)	17.9(15.55∼22.45)	**<0.001**^c^
TG	2.0295(1.50∼3.00)	0.895(0.67∼1.08)	**<0.001**^c^
TC	4.8115(4.36∼5.42)	4.29(3.92∼4.75)	**<0.001**^c^
FBS	5.35(4.80∼5.63)	4.77(4.55∼5.00)	**<0.001**^c^
UA	372(318.75∼455.75)	277(229.75∼342.00)	**<0.001**^c^

**Notes.**

Comparison between two groups (control vs. NAFLD patients). Data represents *n*, mean ±standard deviation or median(range). Bold font format indicates statistical significance.

a Chi-squared tests.

b Two sample Student’s *t*-test.

c Mann–Whitney *U* test.

Abbreviations: ALT alanine aminotransferase AST aspartate aminotransferase TG triglyceride TC total cholesterol FBS fasting blood sugar UA unic acid

### Multivariate analysis of differences between the nafld and control groups

**Figure 2:**
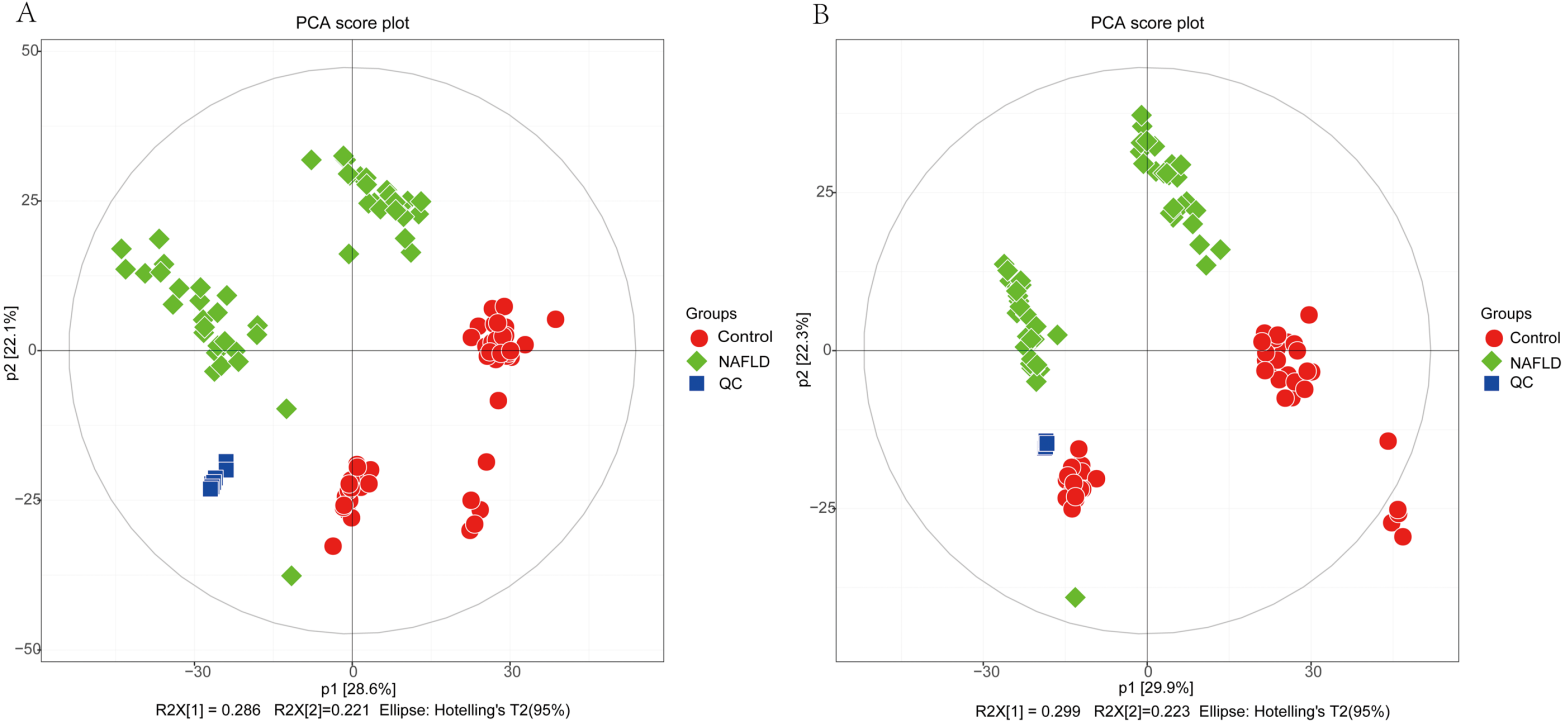
The PCA score plot of 50 healthy controls (red spots) and 50 NAFLD patients (green squares). (A) ESI+; (B) ESI−.

**Figure 3:**
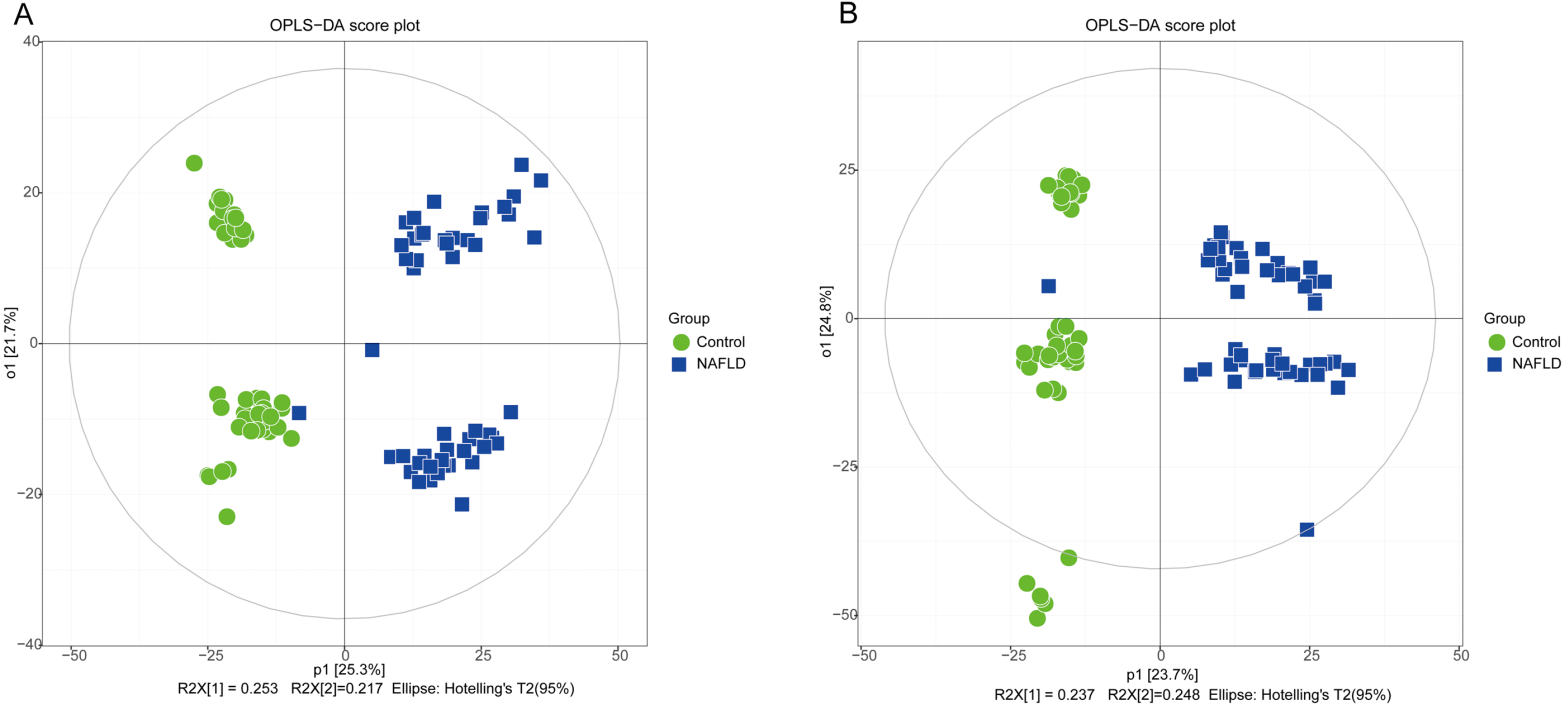
The OPLS-DA score plot of 50 healthy controls (green spots) and 50 NAFLD patients (blue squares). (A) ESI+; (B) ESI−.

The matrix of detected peaks obtained using XCMS was used to perform a multivariate statistical analysis to detect the differences between the NAFLD and control groups. A total of 1,645 variables were included in the matrix in the positive ion mode and 1,463 variables were included in the negative ion mode. Then, the matrix was imported into SIMCA, and all variables were analyzed by unsupervised PCA to determine the general relationships between the two groups, as shown in Figs. 2A and 2B (R2X = 0.507, Q2 = 0.475 in the positive ion mode; R2X = 0.521, Q2 = 0.477 in the negative ion mode), revealing a clear separation trend. Then, orthogonal partial least squares-discriminant analysis (OPLS-DA) was performed, and the results indicated significant separations with valid model fitting (R2X = 0.47, R2Y = 0.899, Q2 = 0.867 in the positive ion mode; R2X = 0.485, R2Y = 0.882, Q2Y = 0.862 in the negative ion mode), as shown in Figs. 3A and 3B. The parameters of the OPLS model indicated that these results were able to reliably and predictably discriminate between the two groups because the R2Y values in the ESI+ and ESI− modes were >0.4. The model did not have an overfitting problem because the R2Y and Q2 values were high and the differences between the R2Y and Q2 values were lower than 0.2.

PCA showed a trend of separation of the groups on the score plot and was able to detect and exclude some outliers, which were defined as observations located outside the 95% confidence region of the model. In our study, NAFLD patients were clearly separated from the healthy controls. Moreover, OPLS-DA models indicated clear separations between the NAFLD and healthy control groups.

### Identification of metabolites in the altered profiles

**Figure 4:**
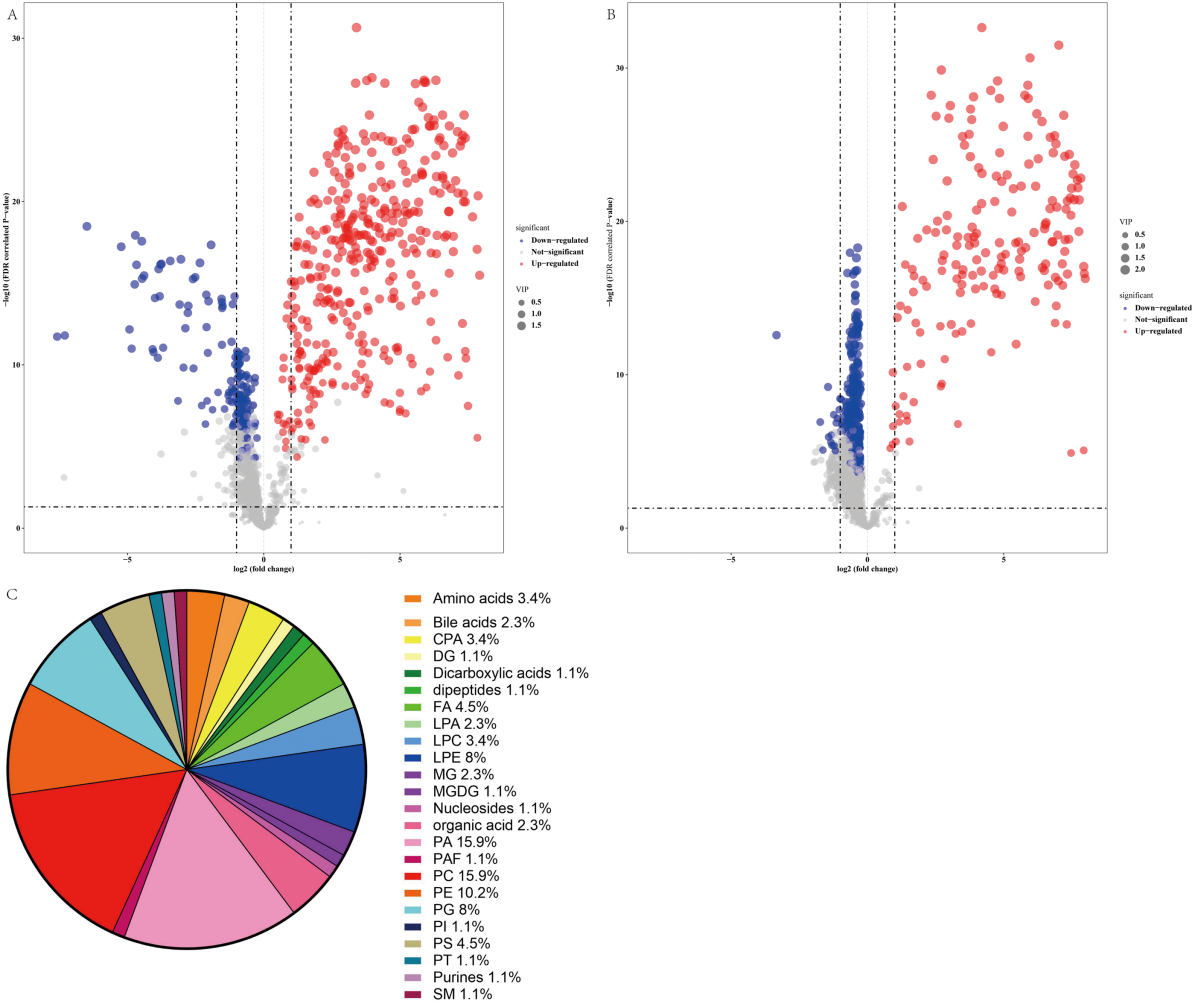
Comparative serum metabolomic profiles of NAFLD patients and healthy controls. (A) and (B) Volcano plot (−log10 (*P* value] and log2 (fold change)) of the features of serum metabolite ions of NAFLD patients and healthy controls. (A) ESI+; (B) ESI −; (C) Percentage of metabolite classes that are significantly different in the serum of NAFLD patients compared with those in the healthy controls. Abbreviations: PA, phosphatidic acid; PC, phosphatidylcholine; PE, phosphatidylserine; LPE, lysophosphatidylethanolamine; PG, phosphatidylglycerol; PS, phosphatidylserine; FA, fatty acyl; CPA, cyclic phosphatidic acid; LPA, lysobisphosphatidic acid; LPC, lysophosphatidylcholine; PI, phosphatidylinositol; and SM, sphingomyelin.

Thus, a panel of 89 variables significantly discriminated the NAFLD and healthy control groups (FDR <0.05), and the volcano plot (Figs. 4A and 4B) showed alterations in 53 metabolites in the ESI+ mode and 41 metabolites in the ESI− mode in serum from NAFLD patients. Furthermore, the serum concentrations of 55 metabolites were increased and the serum concentrations of 39 metabolites were decreased in NAFLD patients compared to those in healthy controls. The families of the changed metabolites contained glycerophospholipids, including phosphatidic acid (PA), phosphatidylcholines (PC), phosphatidylethanolamines (PE), phosphatidylserine (PS), lysophosphatidylethanolamine (LPE), phosphatidylglycerol (PG), cyclic phosphatidic acid (CPA), lysophosphatidylcholines (LPC), lysobisphosphatidic acids (LPA), phosphatidylinositol (PI), fatty acyls, amino acids, bile acids, organic acids, sphingomyelins, dipeptides, purines, and other metabolites. Analysis of all 89 variables indicated that the most abundant altered metabolite families were phosphatidylcholine ≈ phosphatidic acid >phosphatidylethanolamines >lysophosphatidylethanolamine >phosphatidylglycerol, as shown in Fig. 4C.

The error between the qualitative estimate of the compound and the actual molecular weight of the compound was described by Δppm. Metabolites with m/z within 5 ppm and retention time (RT) within 50 min were selected for further study. In total, 35 metabolites were accurately recognized based on this standard, as summarized in Table 2. Analysis of these metabolites indicated that the contents of amino acids, including L-homocitrulline and N-succinyl-L-diaminopimelic acid, were increased in the NAFLD group. The level of glutaconic acid, which was classified as dicarboxylic acid, was increased in the NAFLD group. The levels of fatty acid esters, including L-acetylcarnitine and propionylcarnitine, were increased in the NAFLD group. The changes in glycerophospholipids were variable because of diverse types of fatty acyl chains. Additionally, the changes in fatty acids and their conjugates were variable. The level of 2-isopropylmalic acid was decreased in the NAFLD group, and the level of 20-COOH-leukotriene B4 was increased in the NAFLD group. Table 3 shows the metabolites with higher area under the curve (AUC). Analysis of these metabolites indicated that L-acetylcarnitine, L-homocitrulline, and glutamic acid were the top 3 metabolites ranked by VIP score (VIP 1.9, 1.94, 1.9, respectively) and had favorable effective functions (AUC 0.9952, 95% CI [0.985–1.000]; 0.9908, 95% CI [0.966–1.000]; 0.9884 95% CI [0.973–1.000], respectively, *p* < 0.001) for diagnosis.

### Pathway analysis of altered profiles

**Figure 5:**
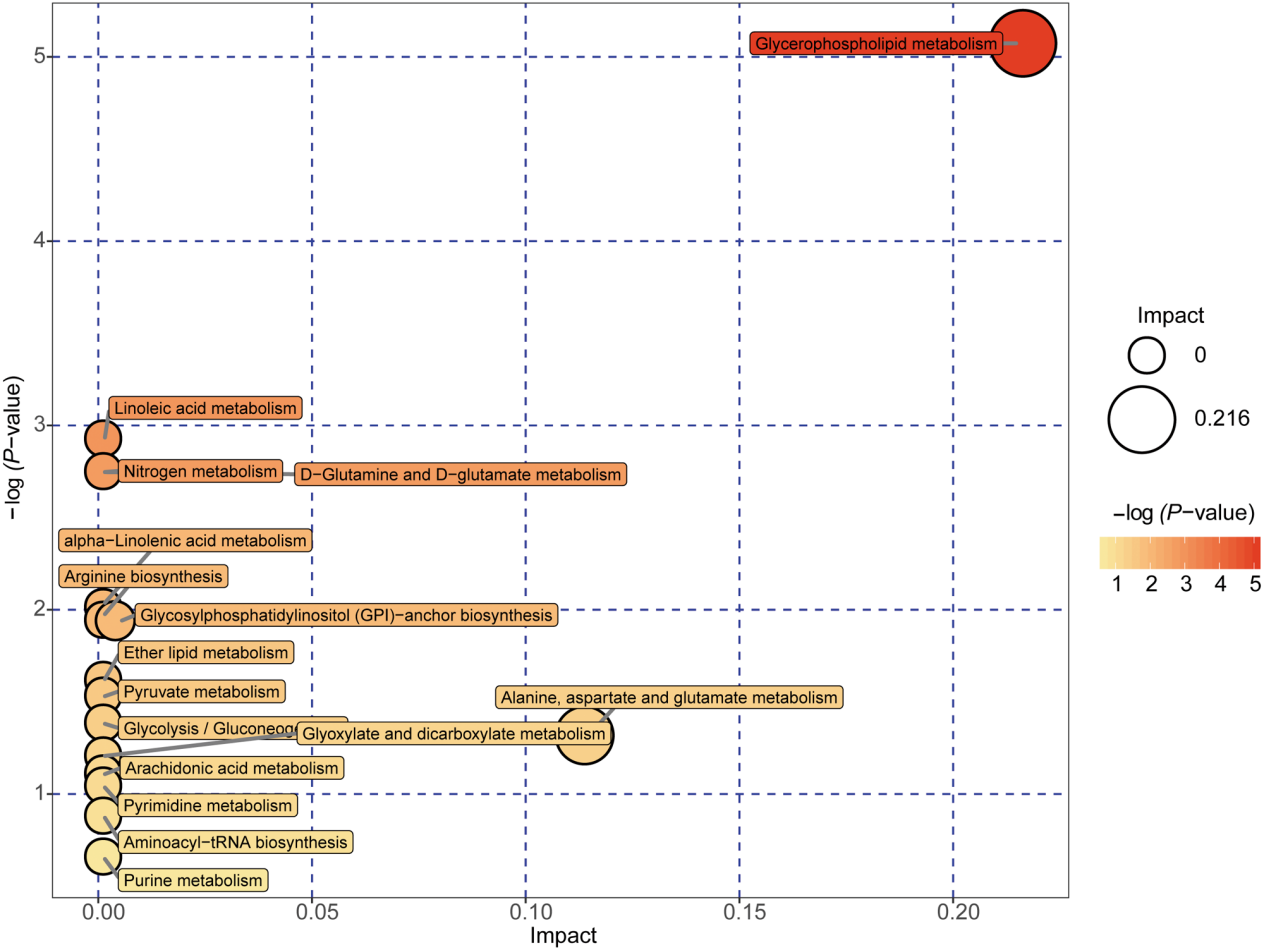
Metabolome map of metabolic pathways relevant for the changes in the serum metabolic profiles of NAFLD patients versus healthy controls. Colors varying from yellow to red indicate metabolites detected in the present study with different levels of significance according to the enrichment analysis. The original *p* values were calculated based on the enrichment analysis, and the impact values indicate the pathway impact values calculated based on the pathway topology analysis.

A total of 89 metabolites altered in the NAFLD group versus healthy control group were selected for metabolomic pathway analysis (MetPA). The relevant pathways for the NAFLD patients and healthy controls were visualized by an interactive visualization framework in Fig. 5. Metabolic pathways with the impact values >0.1 or −log(p) >10 was considered the most relevant pathways involved in the studied conditions [10]. In the present study, 14 metabolic pathways were selected as potential metabolic pathways for NAFLD patients and healthy controls based on their impact value, as shown in Table 4. In these pathways, some biological modules were involved in the lipid metabolism, including glycerophospholipid metabolism, linoleic acid metabolism, alpha-linolenic acid metabolism, and ether lipid metabolism. Some biological modules were involved in the carbohydrate metabolism, including pyruvate metabolism, glycolysis/gluconeogenesis, and glyoxylate and dicarboxylate metabolism. Glycerophospholipid metabolism was the most relevant pathway.

**Table 2:** Qualitative identification results of differential serum metabolites in NAFLD.

No.	Retention time (min)	Ion mode	Query	VIP	Δppm	FDR-PV	FC	Direction of viration	Class
1	2.13	ESI−	Glutaconic acid	1.94	4	3.16154E−24	7.11	↑	Dicarboxylic acids and derivatives
2	6.17	ESI+	L-Homocitrulline	1.9	2	9.65529E−24	6.71	↑	Amino acids, peptides, and analogues
3	5.14	ESI+	L-Acetylcarnitine	1.9	2	1.02404E−25	20.06	↑	Fatty acid esters
4	7.86	ESI−	LysoPE(0:0/22:1)	1.9	1	5.19367E−25	52.85	↑	Glycerophosphoethanolamines
5	12.73	ESI−	PE(18:3/20:5)	1.87	0	2.34822E−21	105.90	↑	Glycerophosphoethanolamines
6	6.75	ESI+	N-Succinyl-L-diaminopimelic acid	1.86	0	5.78078E−28	14.70	↑	Amino acids, peptides, and analogues
7	12.30	ESI+	PE(14:0/14:0)	1.85	3	1.80239E−22	4.53	↑	Glycerophosphoethanolamines
8	12.81	ESI+	PC(14:1/22:6)	1.84	5	3.25029E−21	4.38	↑	Glycerophosphocholines
9	12.17	ESI−	PE(18:4/18:4)	1.8	0	7.63936E−19	17.19	↑	Glycerophosphoethanolamines
10	12.47	ESI−	PA(20:3/20:5)	1.79	3	3.5061E−20	2.84	↑	Glycerophosphates
11	13.27	ESI−	PE(20:4/20:4)	1.74	3	6.87857E−17	2.66	↑	Glycerophosphoethanolamines
12	12.46	ESI+	PA(13:0/17:1))	1.72	1	7.21129E−21	2.44	↑	Glycerophosphates
13	12.90	ESI+	PE(18:/22:6)	1.72	0	2.22908E−18	4.54	↑	Glycerophosphoethanolamines
14	13.06	ESI−	MGDG(18:3/18:4)	1.62	0	3.61325E−17	2.28	↑	
15	3.42	ESI+	2-Isopropylmalic acid	1.49	0	1.26439E−14	0.38	↓	Fatty acids and conjugates
16	14.31	ESI+	PA(18:0/0:0)	1.43	3	6.53123E−14	0.26	↓	Glycerophosphates
17	7.51	ESI+	Propionylcarnitine	1.41	0	5.31064E−15	16.06	↑	Fatty acid esters
18	12.44	ESI+	PS(21:0/0:0)	1.38	2	1.88192E−11	3.07	↑	Glycerophosphoserines
19	10.69	ESI−	LysoPE(18:0/0:0)	1.37	0	2.32189E−13	0.84	↓	Glycerophosphoethanolamines
20	9.22	ESI+	20-COOH-Leukotriene B4	1.33	3	8.06805E−11	2.61	↑	Fatty acids and conjugates
21	12.54	ESI+	PC(16:1/2:0)	1.31	5	1.39377E−11	0.72	↓	Glycerophosphocholines
22	10.28	ESI−	LysoPE(0:0/20:2)	1.28	0	1.97997E−13	0.76	↓	Glycerophosphoethanolamines
23	12.36	ESI−	PT(18:0/18:1)	1.25	1	1.44434E−08	0.80	↓	
24	12.57	ESI+	PC(O-16:0/3:0)	1.18	2	4.14917E−10	0.74	↓	Glycerophosphocholines
25	0.66	ESI−	PC(14:1/16:1)	1.17	4	1.78018E−06	0.80	↓	Glycerophosphocholines
26	12.18	ESI+	PE(16:0/20:4)	1.16	3	1.10602E−07	0.71	↓	Glycerophosphoethanolamines
27	9.54	ESI−	N-palmitoyl-phosphoethanolamine	1.13	2	5.54069E−09	0.68	↓	Organic phosphonic acids
28	10.66	ESI+	LysoPC(18:3)	1.11	2	1.20618E−09	0.86	↓	Glycerophosphocholines
29	11.19	ESI+	PC(O-16:0/5:0)	1.1	5	2.78952E−07	0.70	↓	Glycerophosphocholines
30	8.46	ESI+	C16 Sphinganine	1.09	2	7.55376E−09	0.53	↓	Phosphosphingolipids
31	0.67	ESI−	Purine	1.08	0	0.000016678	0.83	↓	Purines and purine derivatives
32	11.92	ESI−	PC(O-1:0/16:0)	1.07	0	5.24049E−08	0.84	↓	Glycerophosphocholines
33	9.58	ESI+	PA(21:4/0:0)	1.05	3	1.19698E−06	1.63	↑	Glycerophosphates
34	11.24	ESI+	PC(O-1:0/16:0)	1.04	3	1.49395E−08	0.74	↓	Glycerophosphocholines
35	11.01	ESI−	PC(17:1(10)/0:0)	1.01	1	5.30124E−08	0.81	↓	Glycerophosphocholines

**Notes.**

VIP variable importance in projection FC fold change calculated as the ratio of the mean values in NAFLD patients to that in the controls PV corresponds to *P* value obtained from Student’s *t*-test

Δ ppm corresponds to the error between the qualitative estimate of a compounds and the actual compound that was calculated according to the equation: (exact molecular weight of the compound to be determined - exact molecular weight of the composition of all elements of the actual compound)/exact molecular weight of the composition of all elements of the actual compound*10000.

Direction of variation means the direction of the changed metabolites of fatty liver group compared with the normal group.

Compounds were confirmed by reference standards.

**Table 3:** Diagnostic capacity of top 20 metabolites ranked by the AUC value.

Metabolite	Ion mode	AUC	SEN(%)	SPE(%)	VIP	Fold change
PE(14:0/14:0)	ESI+	0.998	1	0.96	1.85	4.53
L-Acetylcarnitine	ESI+	0.9952	0.98	1	1.9	20.06
N-Succinyl-L-diaminopimelic acid	ESI+	0.9928	0.98	1	1.86	14.70
PC(14:1/22:6)	ESI+	0.9928	0.98	1	1.84	4.38
Glutaconic acid	ESI−	0.9908	0.98	1	1.94	7.11
L-Homocitrulline	ESI+	0.9884	0.98	1	1.9	6.71
PE(18:3/20:5)	ESI−	0.988	0.98	1	1.87	105.90
LysoPE(0:0/22:1)	ESI−	0.9876	0.98	1	1.9	52.85
PE(18:4/18:4)	ESI−	0.9844	0.98	1	1.8	17.19
PE(20:4/20:4)	ESI−	0.984	0.94	0.98	1.74	2.66
PA(20:3/20:5)	ESI−	0.984	0.96	1	1.79	2.84
Propionylcarnitine	ESI+	0.9812	0.98	1	1.41	16.06
PE(18:/22:6)	ESI+	0.972	0.98	0.98	1.72	4.54
PA(13:0/17:1))	ESI+	0.9716	1	0.9	1.72	2.44
MGDG(18:3/18:4)	ESI−	0.9536	0.94	0.9	1.62	2.28
PS(21:0/0:0)	ESI+	0.9524	0.92	0.9	1.38	3.07
20-COOH-Leukotriene B4	ESI+	0.8924	0.96	0.78	1.33	2.61

**Notes.**

Abbreviations: AUC area under the receiver operating characteristic curve SEN sensitivity SPE specificity VIP variable importance in projection

**Table 4:** Detailed results of potential metabolic pathways of NAFLD patients and healthy controls.

Potential metabolic pathway	−log(p)	Impact	Relevant metabolites
Glycerophospholipid metabolism	5.0737	0.21631	Phosphatidylethanolamine Phosphatidylcholine 1-Acyl-sn-glycero-3 phosphocholine
Linoleic acid metabolism	2.924	0	Phosphatidylcholine
D-Glutamine and D-glutamate metabolism	2.7469	0	L-Glutamine
Nitrogen metabolism	2.7469	0	L-Glutamine
alpha-Linolenic acid metabolism	2.0095	0	Phosphatidylcholine
Arginine biosynthesis	1.9405	0	L-Glutamine
Glycosylphosphatidylinositol (GPI)-anchor biosynthesis	1.9405	0.00399	Phosphatidylethanolamine
Ether lipid metabolism	1.6143	0	1-(1-Alkenyl)-sn-glycero-3-phosphate
Pyruvate metabolism	1.5291	0	(S)-Lactate
Glycolysis/Gluconeogenesis	1.3821	0	(S)-Lactate
Alanine, aspartate and glutamate metabolism	1.318	0.11378	L-Glutamine
Glyoxylate and dicarboxylate metabolism	1.2044	0	L-Glutamine
Arachidonic acid metabolism	1.1064	0	Phosphatidylcholine
Pyrimidine metabolism	1.0411	0	L-Glutamine

**Notes.**

−log(p), the original *P* value calculated based on the enrichment analysis. Impact, the pathway impact value calculated based on the pathway topology analysis.

## Discussion

NAFLD is a multifactorial disease. The pathogenic factors include genetic factors, the environment, metabolic disturbances, and other factors that may be induced by acquired metabolic stress. The pathogenesis of NAFLD remains unclear and is associated with genetic susceptibility. On the other hand, NAFLD is closely associated with metabolic disorders. The metabolic features of NAFLD may vary because of racial and ethnic factors linked to differences in genetics and diet.

The metabolic profiles of NAFLD patients in the present study were completely different from the profiles of healthy controls. The contents of the majority of the altered metabolites were increased in the NAFLD group. The most abundant altered metabolite families were mainly glycerophospholipids, including PC, PA, PE, and PG. A serum metabolomic study of patients with hyperuricemia demonstrated similar results indicating that the progression of NAFLD in patients with hyperuricemia was associated with disturbances in the phospholipase metabolism (Tan et al., 2016). According to the pathway enrichment analysis, glycerophospholipid metabolism had the closest relationship. Several animal studies have shown that some protective effects of medications, such as Shengling Baizhu San and total turmeric extract, and genetic factors, i.e., growth arrest and DNA damage-inducible protein 45 *α*, in the animal models of NAFLD target the glycerophospholipid metabolism pathway (Deng et al., 2019; Tanaka et al., 2017; Wang et al., 2016). Glycerophospholipid metabolism is complex, and the changes in glycerophospholipids detected in our study are variable. Various PEs were increased or decreased in the NAFLD group compared to those in the healthy control group; however, most PEs were increased in the NAFLD group. Similar results were obtained in the case of PC. Abnormally high or low levels of PC or PE can influence energy metabolism (van der Veen et al., 2017). Some animal studies reported that the turnover of PC and PE species was increased in the liver in the animal models of NAFLD/NASH (Hyde et al., 2009; van Ginneken et al., 2007; Vinaixa et al., 2010).

This study is the first to report an increase in L-homocitrulline in the NAFLD group compared to that in the healthy controls. Homocitrulline is derived by carbamylation. Carbamylation is one of the posttranslational modifications that change the structure and function of proteins. Carbamylated proteins are known to be associated with various diseases, such as atherosclerosis (Jaisson et al., 2015; Speer et al., 2014; Sun et al., 2016), autoimmune disease (Pruijn, 2015), chronic kidney disease (CKD) (Jaisson et al., 2018), thrombus formation(Holy et al., 2016), and infections (Koro et al., 2014). Some metabolomic studies have shown a correlation between the levels of homocitrulline and other diseases. A clinical trial in Germany showed that homocitrulline was significantly associated with the causes of CKD (Grams et al., 2017). Another metabolite analysis showed that homocitrulline was progressively increased during the development of Alzheimer’s dementia (Corso et al., 2017). A cross-sectional study on children with environmental enteric dysfunction in the USA reported that homocitrulline was positively associated with gut permeability (Semba et al., 2017). Interestingly, plasma metabolomic analysis of patients with alcoholic hepatitis (AH) detected significantly higher levels of homocitrulline in the alcoholic hepatitis groups and demonstrated that the plasma levels of homocitrulline were correlated with the Model for End-stage Liver Disease (MELD) scores in AH patients (Ascha et al., 2016). However, only a few studies investigated homocitrulline and carbamylation in NAFLD. Carbamylation is a nonenzymatic reaction with isocyanic acid. Isocyanic acid has two main origins, one of which is urea deamination. Ornithine transcarbamylase (OTC) and carbamoyl phosphate synthetase (CPS1), which are enzymes involved in the urea cycle, are present in the mitochondria, and mitochondrial dysfunction is associated with the progression of NAFLD (Pessayre & Fromenty, 2005). Another origin of isocyanic acid is thiocyanate oxidation by myeloperoxidase (MPO), which often occurs under inflammatory conditions and in atherosclerotic plaques. MPO is present in some immunocytes, including monocytes, neutrophils, and certain tissue macrophages (Odobasic, Kitching & Holdsworth, 2016). These phenomena indicate a possible link between carbamylation and NAFLD.

Pathway enrichment analysis performed in the present study suggested 14 potential differential metabolic pathways in NAFLD patients and healthy controls based on their impact value. Biological modules involved in lipid metabolism and carbohydrate metabolism were the most relevant to NAFLD. Insulin-sensitizing thiazolidinedione compounds can treat NASH by binding and inhibiting the mitochondrial pyruvate carrier (Colca, 2020)[41]. A study on the serum metabolomic biomarkers of NAFLD in Iranian patients showed elevated levels of the TCA cycle intermediates in NAFLD patients compared to those in healthy controls (Chashmniam et al., 2019)[42]. In summary, both studies highlighted the role of mitochondrial dysfunction in the progression of NAFLD.

## Conclusions

Overall, this study identified significant alterations in the metabolic profiles of NAFLD patients versus healthy controls. The metabolic profiles of Chinese NAFLD patients were characterized by alterations in glycerophospholipids, and pathway enrichment analysis demonstrated that glycerophospholipid metabolism was the most closely related metabolic pathway. L-Homocitrulline, which is a carbamylation-derived metabolite, was remarkably increased in NAFLD patients. This study has some limitations. First, this was a retrospective observational study that could not show a causal link between metabolites and NAFLD. Second, additional experiments are required to confirm the associations between homocitrulline, NAFLD, and the metabolic pathways.

##  Supplemental Information

10.7717/peerj.11346/supp-1 Supplemental Information 1 The anonymised numeric raw data and normalized data of the metabolomic profiles between NAFLD patients and healthy controls in ESI+ mode The data were analyzed using XCMS software for peak picking, peak alignment > peak filtering and peak filling. The data were normalized using Excel 2010, including Retention time (RT), MZ, Observations (samples) and peak intensity

10.7717/peerj.11346/supp-2 Supplemental Information 2 The anonymised numeric raw data and normalized data of the metabolomic profiles between NAFLD patients and healthy controls in ESI− mode The data were analyzed using XCMS software for peak picking, peak alignment > peak filtering and peak filling. The data were normalized using Excel 2010, including Retention time (RT), MZ, Observations (samples) and peak intensity

10.7717/peerj.11346/supp-3 Supplemental Information 3 The anonymised raw numeric clinical data including group, gender, age, ALT, AST, etc

10.7717/peerj.11346/supp-4 Supplemental Information 4 The gradient of mobile phase

10.7717/peerj.11346/supp-5 Supplemental Information 5 The significantly different metabolites in A-B at (ESI+)

10.7717/peerj.11346/supp-6 Supplemental Information 6 The significantly different metabolites in A–B at (ESI−)

10.7717/peerj.11346/supp-7 Supplemental Information 7 The Total Ion Chromatogram of QC samples, (A) ESI+, (B) ESI−

## Additional Information and Declarations

### Data Availability

The following information was supplied regarding data availability: The raw measurements are available in the Supplemental Files.
