# Supplementary material for: Serum metabolomic profiling reveals an increase in homocitrulline in Chinese patients with nonalcoholic fatty liver disease: a retrospective study
Source: PeerJ. 2021 May 3;9:e11346. doi: 10.7717/peerj.11346 (PMC8101472; doi:10.7717/peerj.11346)
Supplement: Supplemental Information 4 [file peerj-09-11346-s004.docx]

Supplemental Table 1. The gradient of mobile phase

| **Time(min)** | **Flow(mL/min)** | **Pressure Limit(bar)** | **Solv Ratio B(%)** |
| --- | --- | --- | --- |
| 0 | 0.35 | 800 | 5 |
| 1 | 0.35 | 800 | 5 |
| 6 | 0.35 | 800 | 20 |
| 9 | 0.35 | 800 | 50 |
| 13 | 0.35 | 800 | 95 |
| 15 | 0.35 | 800 | 95 |
