# Supplementary material for: Serum metabolomic profiling reveals an increase in homocitrulline in Chinese patients with nonalcoholic fatty liver disease: a retrospective study
Source: PeerJ. 2021 May 3;9:e11346. doi: 10.7717/peerj.11346 (PMC8101472; doi:10.7717/peerj.11346)
Supplement: Supplemental Information 5 [file peerj-09-11346-s005.docx]

**Supplementary Table 2. The Significantly different metabolites in A-B at (ESI+)**

| ID | VIP | mz | Δppm | Identify | Formula | Mass | RT (min) | pvalue.A_vs_B | fdr.A_vs_B | logfc.A_vs_B |
| --- | --- | --- | --- | --- | --- | --- | --- | --- | --- | --- |
| 42 | 1.18 | 113.0239 | 26 | L-Lactic acid | C3H6O3 | 90.0317 | 0.95 | 1.42588E-07 | 4.37605E-07 | 0.341863542 |
| 191 | 1.49 | 177.0759 | 0 | 2-Isopropylmalic acid | C7H12O5 | 176.0685 | 3.42 | 2.20596E-15 | 1.26439E-14 | 1.404947698 |
| 224 | 1.90 | 190.1191 | 2 | L-Homocitrulline | C7H15N3O3 | 189.1113 | 6.17 | 3.28691E-25 | 9.65529E-24 | -2.746227333 |
| 253 | 1.90 | 204.1232 | 2 | L-Acetylcarnitine | C9H17NO4 | 203.1158 | 5.14 | 1.24504E-27 | 1.02404E-25 | -4.326255636 |
| 286 | 1.41 | 218.1393 | 0 | Propionylcarnitine | C10H19NO4 | 217.1314 | 7.51 | 8.84569E-16 | 5.31064E-15 | -4.005795972 |
| 341 | 1.89 | 242.1085 | 20 | 5-Methyldeoxycytidine | C10H15N3O4 | 241.1063 | 5.55 | 2.80249E-27 | 2.19529E-25 | -4.328990406 |
| 350 | 1.54 | 245.1528 | 13 | Hydroxyprolyl-Isoleucine | C11H20N2O4 | 244.1423 | 5.03 | 5.05148E-19 | 4.19681E-18 | -1.090626126 |
| 422 | 1.09 | 274.2748 | 2 | C16 Sphinganine | C16H35NO2 | 273.2668 | 8.46 | 2.09393E-09 | 7.55376E-09 | 0.905169159 |
| 465 | 1.86 | 291.1185 | 0 | N-Succinyl-L-diaminopimelic acid | C11H18N2O7 | 290.1114 | 6.75 | 3.86557E-30 | 5.78078E-28 | -3.877417937 |
| 600 | 1.33 | 367.2102 | 3 | 20-COOH-Leukotriene B4 | C18H32O6 | 366.2042 | 9.22 | 1.87355E-11 | 8.06805E-11 | -1.381551765 |
| 623 | 1.23 | 380.2562 | 7 | N-palmitoyl-phosphoethanolamine | C18H38NO5P | 379.2488 | 9.57 | 1.7693E-10 | 6.97963E-10 | 0.377392618 |
| 652 | 1.50 | 393.2333 | 17 | CPA(16:0) | C19H37O6P | 392.2328 | 12.96 | 4.61692E-15 | 2.51485E-14 | 1.918750943 |
| 656 | 1.25 | 395.2486 | 18 | LPA(P-16:0e/0:0) | C19H39O6P | 394.2484 | 12.95 | 3.48155E-11 | 1.46101E-10 | 2.040711251 |
| 658 | 1.31 | 396.2511 | 10 | PA(15:0/0:0) | C18H37O7P | 396.2277 | 12.93 | 8.2537E-12 | 3.64982E-11 | 2.693747333 |
| 663 | 1.01 | 398.2377 | 18 | PC(9:0/0:0) | C17H36NO7P | 397.2229 | 8.36 | 1.74895E-06 | 4.82722E-06 | -0.564572208 |
| 666 | 1.55 | 399.2812 | 14 | Allolithocholic acid | C24H40O3 | 376.2977 | 10.10 | 1.14513E-15 | 6.77604E-15 | -1.426916518 |
| 726 | 1.60 | 425.2604 | 13 | MG(22:6/0:0/0:0) | C25H38O4 | 402.2770 | 14.02 | 4.7057E-18 | 3.43828E-17 | 2.107202679 |
| 727 | 1.70 | 426.2189 | 14 | PC(5:0/5:0) | C18H36NO8P | 425.2179 | 8.80 | 1.27912E-22 | 2.19183E-21 | -1.739651697 |
| 742 | 1.70 | 429.2487 | 29 | 7-ketodeoxycholic acid | C24H38O5 | 406.2719 | 8.94 | 2.1095E-18 | 1.58454E-17 | -4.4028578 |
| 755 | 1.12 | 433.2428 | 18 | PA(18:3/0:0) | C21H37O7P | 432.2277 | 9.70 | 1.75644E-09 | 6.37825E-09 | 0.599249234 |
| 766 | 1.86 | 439.2307 | 19 | CPA(18:2/0:0) | C21H37O6P | 416.2328 | 9.14 | 2.77443E-23 | 5.3069E-22 | -4.877669421 |
| 769 | 1.43 | 439.2833 | 3 | PA(18:0/0:0) | C21H43O7P | 438.2746 | 14.31 | 1.22684E-14 | 6.53123E-14 | 1.93110006 |
| 775 | 1.88 | 443.2647 | 25 | CPA(18:0/0:0) | C21H41O6P | 420.2641 | 8.48 | 9.85053E-24 | 2.07745E-22 | -4.034122636 |
| 789 | 1.08 | 451.3076 | 23 | PA(P-20:0/0:0) | C23H47O6P | 450.3110 | 14.34 | 9.04249E-09 | 3.08608E-08 | 1.577700362 |
| 793 | 1.19 | 453.1914 | 21 | PA(18:4/0:0) | C21H35O7P | 430.2120 | 9.66 | 3.45674E-08 | 1.12601E-07 | -0.402457832 |
| 812 | 1.60 | 462.3069 | 34 | PC(O-12:0/O-1:0) | C21H46NO6P | 439.3063 | 9.93 | 1.80959E-17 | 1.23518E-16 | -1.576871677 |
| 841 | 1.75 | 475.2894 | 15 | PA(19:0/0:0) | C22H45O7P | 452.2903 | 9.67 | 2.51615E-20 | 2.72307E-19 | -2.077314657 |
| 843 | 1.33 | 476.3231 | 25 | PC(O-12:0/O-2:0) | C22H48NO6P | 453.3219 | 11.27 | 8.57316E-11 | 3.47361E-10 | -2.346009067 |
| 858 | 1.86 | 483.2567 | 12 | LysoPA(22:6/0:0) | C25H39O7P | 482.2433 | 8.47 | 3.05191E-25 | 9.12799E-24 | -6.915834076 |
| 876 | 1.05 | 490.2916 | 3 | PA(21:4/0:0) | C24H44NO7P | 489.2861 | 9.58 | 4.04571E-07 | 1.19698E-06 | -0.703671469 |
| 912 | 1.55 | 504.3152 | 13 | LysoPE(0:0/20:3) | C25H46NO7P | 503.3012 | 10.28 | 1.04049E-12 | 5.03411E-12 | -0.841240684 |
| 927 | 1.04 | 510.3572 | 3 | PC(O-1:0/16:0) | C25H52NO7P | 509.3481 | 11.24 | 4.2321E-09 | 1.49395E-08 | 0.427157508 |
| 952 | 1.11 | 518.3230 | 2 | LysoPC(18:3) | C26H48NO7P | 495.3325 | 10.66 | 3.10894E-10 | 1.20618E-09 | 0.225922901 |
| 954 | 1.45 | 518.3704 | 23 | PC(O-1:0/O-16:0) | C25H54NO6P | 495.3689 | 13.71 | 3.63239E-15 | 2.02552E-14 | 2.138729039 |
| 985 | 1.64 | 532.3468 | 17 | LysoPE(20:0/0:0) | C25H52NO7P | 509.3481 | 11.23 | 6.2145E-15 | 3.35175E-14 | -0.815335937 |
| 987 | 1.34 | 532.3775 | 7 | Lyso-PAF C-18 | C-18 C26H56NO6P | 509.3845 | 13.57 | 8.85769E-13 | 4.33658E-12 | -1.098952346 |
| 992 | 1.15 | 534.3474 | 15 | LysoPE(0:0/22:2) | C27H52NO7P | 533.3481 | 10.73 | 7.66059E-08 | 2.43276E-07 | -0.365185868 |
| 1000 | 1.31 | 536.4057 | 5 | PC(16:1/2:0) | C26H50NO8P | 535.3274 | 12.54 | 2.95699E-12 | 1.39377E-11 | 0.4661638 |
| 1006 | 1.18 | 538.3878 | 2 | PC(O-16:0/3:0) | C27H56NO7P | 537.3794 | 12.57 | 1.02909E-10 | 4.14917E-10 | 0.439912516 |
| 1032 | 1.61 | 548.3754 | 6 | LysoPC(20:2) | C28H54NO7P | 547.3638 | 11.29 | 1.03166E-15 | 6.17121E-15 | -0.718656414 |
| 1072 | 1.10 | 566.4148 | 5 | PC(O-16:0/5:0) | C29H60NO7P | 565.4107 | 11.19 | 8.8688E-08 | 2.78952E-07 | 0.50478258 |
| 1080 | 1.38 | 568.3595 | 2 | PS(21:0/0:0) | C27H54NO9P | 567.3536 | 12.44 | 4.11849E-12 | 1.88192E-11 | -1.61646195 |
| 1096 | 1.18 | 576.4090 | 24 | DG(15:0/18:4/0:0) | C30H58NO7P | 575.3951 | 11.24 | 2.30594E-08 | 7.66318E-08 | -3.457442158 |
| 1105 | 1.33 | 580.4274 | 10 | PC(O-12:0/10:0) | C30H62NO7P | 579.4264 | 12.34 | 8.74423E-11 | 3.53422E-10 | 0.577917014 |
| 1170 | 1.49 | 607.4261 | 11 | PA(12:0/17:0) | C32H63O8P | 606.4261 | 11.57 | 1.07851E-16 | 6.87658E-16 | -0.939617413 |
| 1197 | 1.72 | 619.4325 | 1 | PA(13:0/17:1)) | C33H63O8P | 618.4261 | 12.46 | 4.60295E-22 | 7.21129E-21 | -1.28668091 |
| 1227 | 1.49 | 633.4434 | 8 | PA(17:0/14:1) | C34H65O8P | 632.4417 | 12.73 | 7.89529E-14 | 4.04603E-13 | -0.698744919 |
| 1235 | 1.85 | 636.4576 | 3 | PE(14:0/14:0) | C33H66NO8P | 635.4526 | 12.30 | 8.43672E-24 | 1.80239E-22 | -2.178754339 |
| 1346 | 1.16 | 740.5253 | 3 | PE(16:0/20:4) | C41H74NO8P | 739.5152 | 12.18 | 3.38867E-08 | 1.10602E-07 | 0.502295452 |
| 1358 | 1.76 | 762.5001 | 10 | PE(16:1/22:6)) | C43H72NO8P | 761.4996 | 12.41 | 2.78771E-19 | 2.40093E-18 | -1.825327725 |
| 1363 | 1.37 | 769.4961 | 8 | PG(16:1/20:4) | C42H73O10P | 768.4941 | 13.14 | 4.72578E-11 | 1.94348E-10 | -1.192213222 |
| 1370 | 1.84 | 776.5192 | 5 | PC(14:1/22:6) | C44H74NO8P | 775.5152 | 12.81 | 1.97586E-22 | 3.25029E-21 | -2.132225899 |
| 1383 | 1.72 | 790.5386 | 0 | PE(18:/22:6) | C45H76NO8P | 789.5309 | 12.90 | 2.54752E-19 | 2.22908E-18 | -2.181629824 |
