# Supplementary material for: Serum metabolomic profiling reveals an increase in homocitrulline in Chinese patients with nonalcoholic fatty liver disease: a retrospective study
Source: PeerJ. 2021 May 3;9:e11346. doi: 10.7717/peerj.11346 (PMC8101472; doi:10.7717/peerj.11346)
Supplement: Supplemental Information 6 [file peerj-09-11346-s006.docx]

**Supplementary Table 3. The Significantly different metabolites in A-B at (ESI-)**

| ID | VIP | mz | Δppm | Identify | Formula | Mass | RT (min) | pvalue.A_vs_B | fdr.A_vs_B | logfc.A_vs_B |
| --- | --- | --- | --- | --- | --- | --- | --- | --- | --- | --- |
| 9 | 1.06 | 89.0257 | 14 | L-Lactic acid | C3H6O3 | 90.0317 | 0.97151002 | 1.05525E-06 | 3.29174E-06 | 0.400132424 |
| 31 | 1.08 | 119.0363 | 0 | Purine | C5H4N4 | 120.0436 | 0.665795324 | 6.25851E-06 | 1.6678E-05 | 0.272208753 |
| 40 | 1.94 | 129.0199 | 4 | Glutaconic acid | C5H6O4 | 130.0266 | 2.131150188 | 9.9406E-26 | 3.16154E-24 | -2.830422222 |
| 63 | 1.08 | 145.0640 | 14 | L-Glutamine | C5H10N2O3 | 146.0691 | 0.691650292 | 8.75646E-06 | 2.2714E-05 | 0.207705786 |
| 122 | 1.46 | 174.0562 | 23 | Guanidinosuccinic Acid | C5H9N3O4 | 175.0593 | 3.406534626 | 3.73633E-14 | 2.60298E-13 | 2.3123728 |
| 148 | 1.99 | 188.1019 | 11 | L-Homocitrulline | C7H15N3O3 | 189.1113 | 6.151363181 | 5.28369E-25 | 1.51569E-23 | -5.421340351 |
| 187 | 1.20 | 203.0184 | 6 | Oxaloglutarate | C7H8O7 | 204.027 | 0.707700913 | 1.98419E-08 | 8.29389E-08 | 0.423031738 |
| 232 | 1.81 | 223.0747 | 10 | 3-Hydroxy-DL-kynurenine | C10H12N2O4 | 224.0797 | 6.134923004 | 8.06778E-18 | 7.42338E-17 | -4.993416792 |
| 567 | 1.13 | 378.2405 | 2 | N-palmitoyl-phosphoethanolamine | C18H38NO5P | 379.2488 | 9.541193589 | 1.07557E-09 | 5.54069E-09 | 0.564473391 |
| 640 | 1.90 | 415.2703 | 21 | MG(0:0/20:3/0:0) | C21H37O7P | 432.2277 | 10.1782997 | 1.05266E-20 | 1.54004E-19 | -5.208944599 |
| 795 | 1.37 | 480.3092 | 0 | LysoPE(18:0/0:0) | C23H48NO7P | 481.3168 | 10.68726994 | 3.30112E-14 | 2.32189E-13 | 0.25799685 |
| 836 | 1.91 | 502.3087 | 29 | LysoPE(0:0/20:3) | C25H46NO7P | 503.3012 | 10.45641273 | 1.85852E-20 | 2.56511E-19 | -2.724857321 |
| 839 | 1.28 | 504.3091 | 0 | LysoPE(0:0/20:2) | C25H48NO7P | 505.3168 | 10.28416915 | 2.78792E-14 | 1.97997E-13 | 0.391144363 |
| 842 | 1.01 | 506.3244 | 1 | PC(17:1(10)/0:0) | C25H50NO7P | 507.3325 | 11.00741288 | 1.20301E-08 | 5.30124E-08 | 0.308586482 |
| 845 | 1.07 | 508.3409 | 0 | PC(O-1:0/16:0) | C25H52NO7P | 509.3481 | 11.92226617 | 1.18565E-08 | 5.24049E-08 | 0.246105789 |
| 911 | 1.21 | 542.3362 | 20 | LysoPC(20:4) | C28H50NO7P | 543.3325 | 10.68113708 | 7.2711E-11 | 4.20459E-10 | 0.174162185 |
| 923 | 1.13 | 552.3208 | 17 | LysoPE(0:0/24:6) | C29H48NO7P | 553.3168 | 10.45901429 | 1.54864E-11 | 9.47973E-11 | 0.366179899 |
| 938 | 1.05 | 556.2999 | 8 | PC(10:2/10:2) | C28H48NO8P | 557.3118 | 10.30711581 | 3.51373E-08 | 1.42005E-07 | 0.647965683 |
| 959 | 1.07 | 565.3353 | 27 | PG(22:1(11Z)/0:0) | C28H55O9P | 566.3584 | 10.28416915 | 1.8046E-09 | 9.0726E-09 | 0.340416167 |
| 969 | 1.90 | 570.3340 | 1 | LysoPE(0:0/22:1) | C27H54NO7P | 535.3638 | 7.86123059 | 1.42001E-26 | 5.19367E-25 | -5.723891596 |
| 993 | 1.25 | 583.3357 | 17 | PI(P-18:0/0:0) | C27H53O11P | 584.3325 | 11.00741288 | 7.90412E-11 | 4.51708E-10 | 0.231615855 |
| 1126 | 1.78 | 661.4164 | 11 | PG(14:1/14:1) | C34H63O10P | 662.4159 | 12.42648796 | 3.86042E-19 | 4.51824E-18 | -8.569376242 |
| 1129 | 1.75 | 662.4181 | 21 | PS(12:0/15:1) | C33H62NO10P | 663.4111 | 12.42020337 | 3.33166E-18 | 3.24948E-17 | -2.679365252 |
| 1141 | 1.21 | 674.5188 | 8 | PE(14:0/P-18:0) | C37H74NO7P | 675.5203 | 0.669738955 | 7.05626E-08 | 2.65381E-07 | 0.373417633 |
| 1142 | 1.77 | 675.4310 | 9 | PG(12:0/17:2) | C35H65O10P | 676.4315 | 12.80988162 | 2.6363E-17 | 2.32344E-16 | -3.305868931 |
| 1162 | 1.81 | 699.4316 | 10 | PG(13:0/18:4) | C37H65O10P | 700.4315 | 12.36080832 | 5.13797E-18 | 4.84958E-17 | -7.169127862 |
| 1170 | 1.33 | 713.4307 | 12 | PG(12:0/20:4) | C38H67O10P | 714.4472 | 13.47467243 | 1.97645E-08 | 8.28522E-08 | -1.032791123 |
| 1172 | 1.97 | 715.4214 | 18 | PA(18:3/20:5) | C41H65O8P | 716.4417 | 11.19865355 | 1.52385E-26 | 5.43754E-25 | -2.630470464 |
| 1179 | 1.83 | 729.4417 | 11 | PA(17:2/22:6) | C42H67O8P | 730.4574 | 12.17957503 | 2.02332E-21 | 3.442E-20 | -6.328022054 |
| 1180 | 1.80 | 730.4451 | 0 | PE(18:4/18:4) | C41H66NO8P | 731.4526 | 12.17428046 | 5.79609E-20 | 7.63936E-19 | -4.103132933 |
| 1183 | 1.03 | 734.4706 | 8 | PE(14:0/22:6) | C41H70NO8P | 735.4839 | 0.659998831 | 3.86112E-05 | 8.81252E-05 | 0.281622192 |
| 1186 | 1.17 | 736.4656 | 4 | PC(14:1/16:1) | C38H72NO8P | 701.4996 | 0.662528629 | 5.42294E-07 | 1.78018E-06 | 0.324358054 |
| 1188 | 1.11 | 738.4641 | 10 | PS(P-16:0/18:4) | C40H70NO9P | 739.4788 | 0.664424313 | 2.20022E-06 | 6.38676E-06 | 0.315887758 |
| 1191 | 1.79 | 743.4635 | 3 | PA(20:3/20:5) | C43H69O8P | 744.473 | 12.46583161 | 2.10893E-21 | 3.5061E-20 | -1.507603191 |
| 1192 | 1.82 | 744.4664 | 21 | PS(13:0/20:2) | C39H72NO10P | 745.4894 | 12.46730194 | 5.31062E-22 | 9.59191E-21 | -1.780941571 |
| 1205 | 1.87 | 757.4732 | 7 | PG(16:0/16:0) | C38H75O10P | 722.5097 | 12.73618947 | 1.82512E-22 | 3.42326E-21 | -6.749321567 |
| 1206 | 1.87 | 758.4764 | 0 | PE(18:3/20:5) | C43H70NO8P | 759.4839 | 12.73476912 | 1.2038E-22 | 2.34822E-21 | -6.726541428 |
| 1208 | 1.83 | 759.4788 | 6 | PA(18:0/20:4) | C41H73O8P | 724.5043 | 12.73454397 | 1.13331E-20 | 1.64161E-19 | -6.407009143 |
| 1211 | 1.62 | 771.5055 | 0 | MGDG(18:3/18:4) | C45H72O10 | 772.5125 | 13.06391582 | 3.75403E-18 | 3.61325E-17 | -1.186391529 |
| 1217 | 1.74 | 786.5110 | 3 | PE(20:4/20:4) | C45H74NO8P | 787.5152 | 13.27408736 | 7.42867E-18 | 6.87857E-17 | -1.410146224 |
| 1230 | 1.25 | 802.5595 | 1 | PT(18:0/18:1) | C43H82NO10P | 803.5676 | 12.35601397 | 2.98148E-09 | 1.44434E-08 | 0.316861941 |
