# Supplementary figures and images for: Serum metabolomic profiling reveals an increase in homocitrulline in Chinese patients with nonalcoholic fatty liver disease: a retrospective study
Source: PeerJ. 2021 May 3;9:e11346. doi: 10.7717/peerj.11346 (PMC8101472; doi:10.7717/peerj.11346)

A

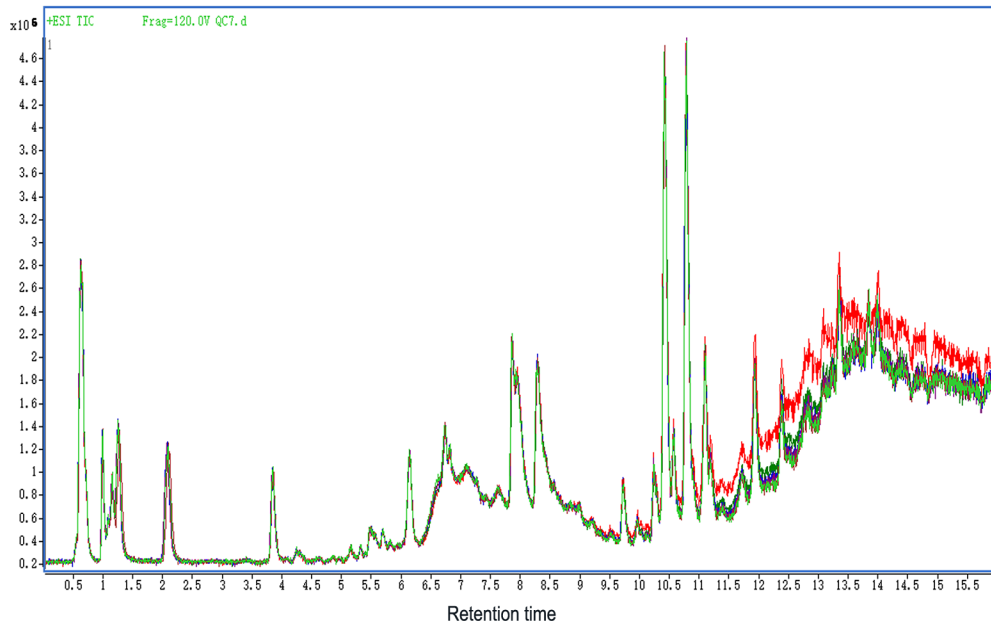

B

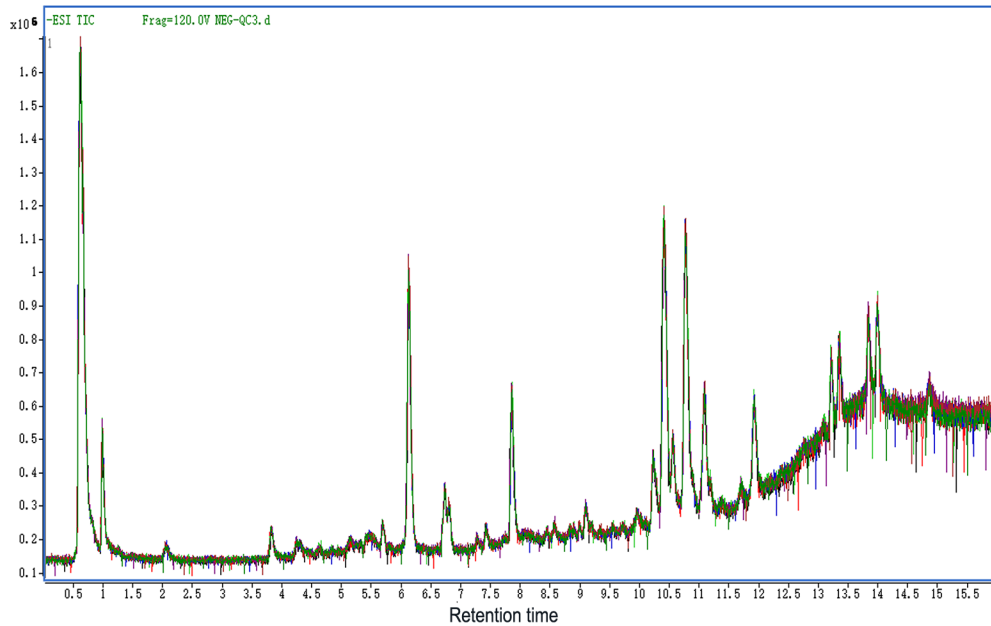

Supplement: Supplemental Information 7 [file peerj-09-11346-s007.pdf]
